# Supplementary material for: Mangrove removal exacerbates estuarine infilling through landscape-scale bio-morphodynamic feedbacks
Source: Nat Commun. 2023 Nov 11;14:7310. doi: 10.1038/s41467-023-42733-1 (PMC10640651; doi:10.1038/s41467-023-42733-1)
Supplement: Supplementary file 1 — Supplementary Information [file 41467_2023_42733_MOESM1_ESM.pdf]

File name: Supplementary Information 1

Description: Supplementary Figures and Supplementary Tables

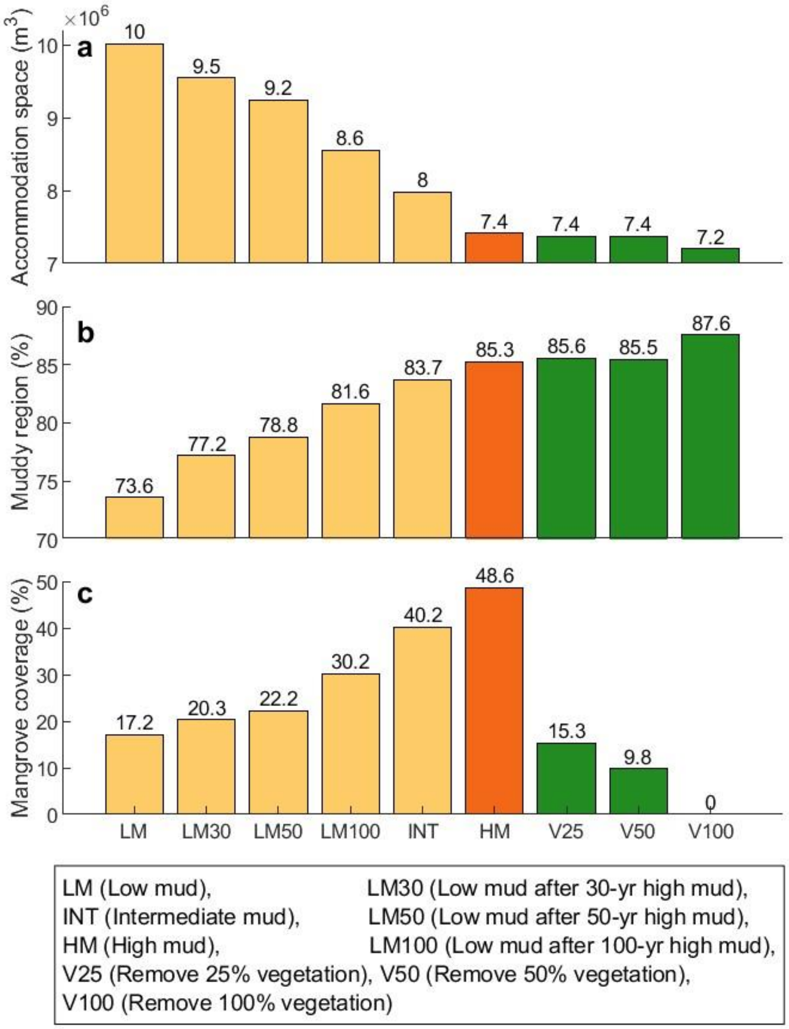

Fig. S1 Key morphological, sedimentological and ecological characteristics of the estuarine environment for scenarios of mud supply and mangrove removal. a) Accommodation space, b) muddy region and c) mangrove coverage.

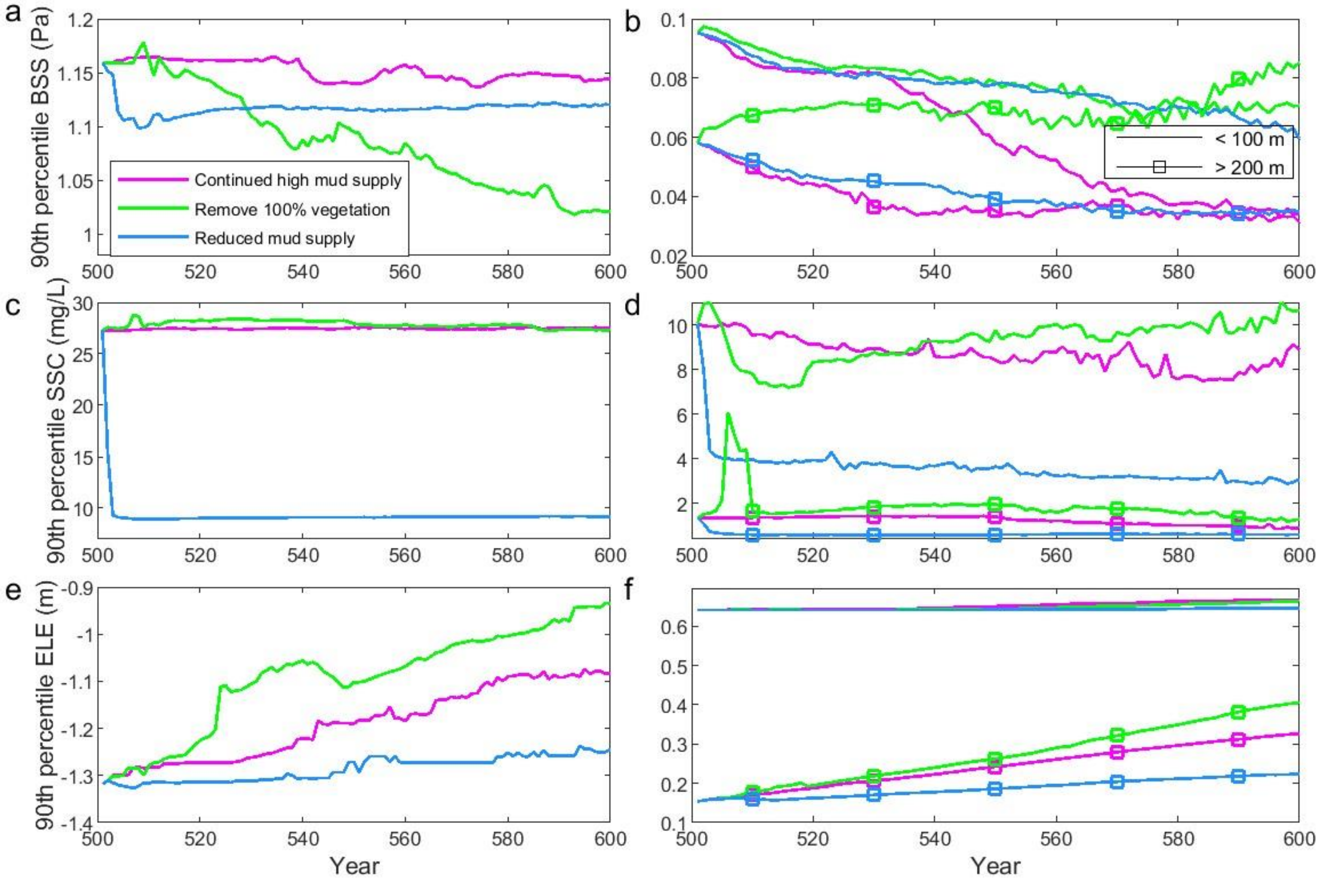

Fig. S2 Temporal changes in 90th percentile bed shear stress (BSS), suspended sediment concentration (SSC) and bed elevation (ELE) in future scenarios. The analysis is based on the southern reach of the system (see Fig. S3) and further categorized as channelized area (a,c&e) and unchannelized area (b,d&f). The unchannelized area is further divided into two regions, with one region close to the channel (< 100 m) and the other region further away from the channel (> 200 m).

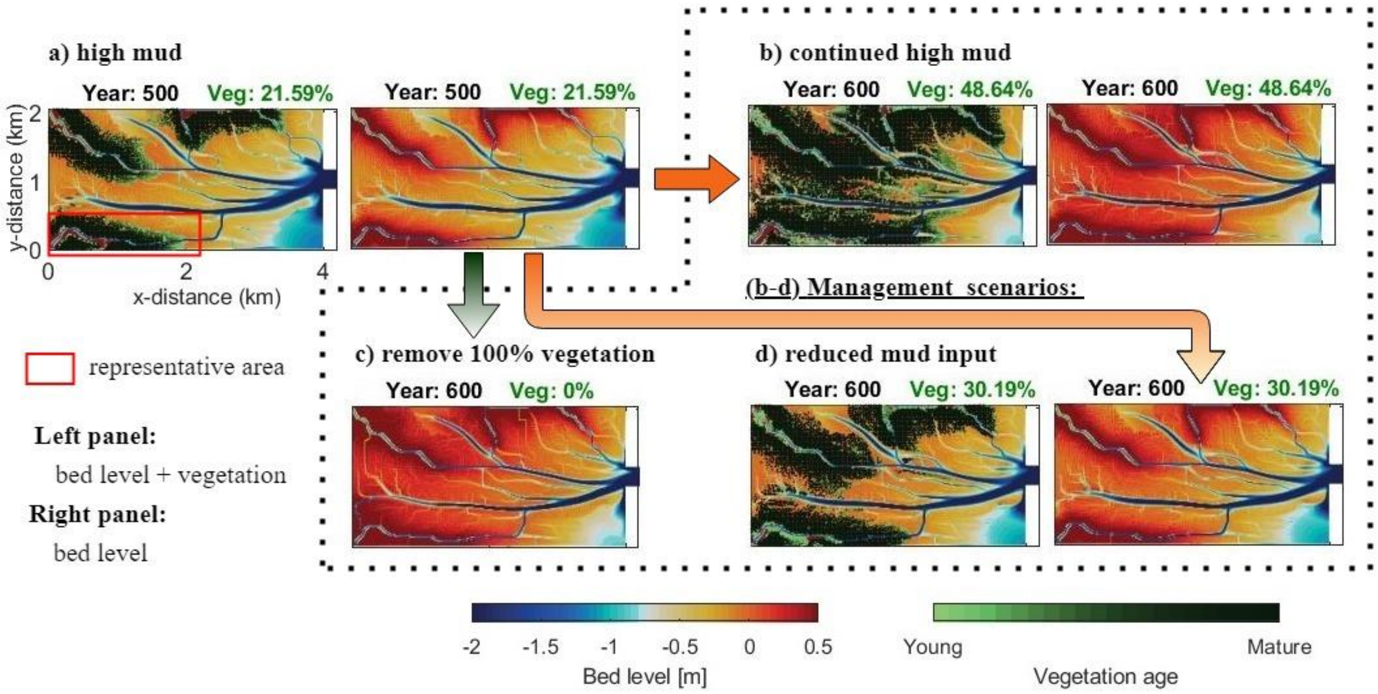

Fig. S3 Representative area for analyzing the changes of parameters in Fig. S2. Subplots a, b & d contain two different panels: the left panel shows the bed level and indicates the presence of vegetation, while the right panel only shows the bed level.

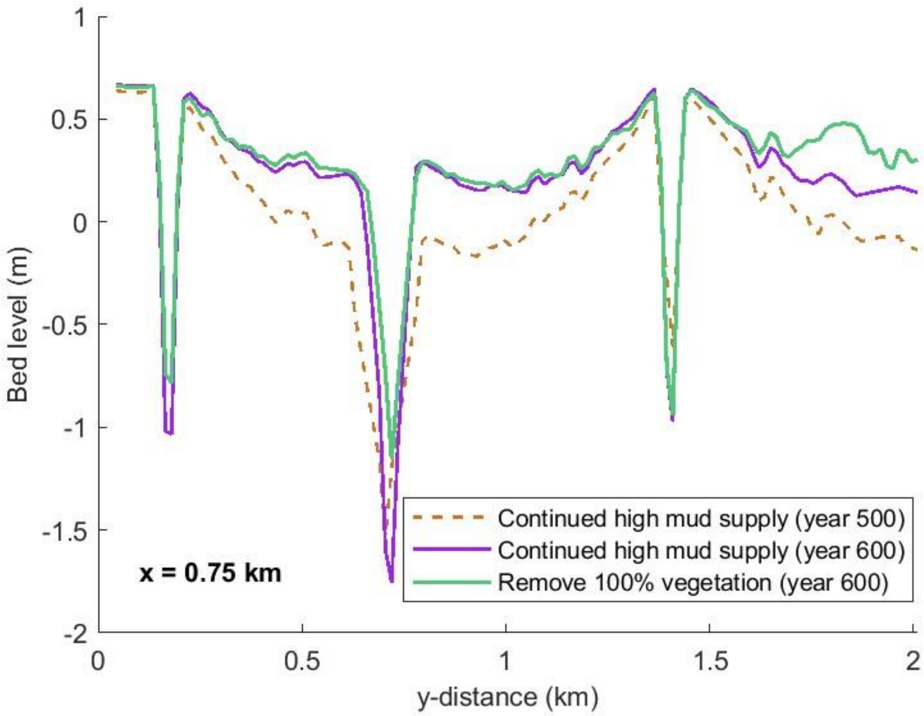

Fig. S4 Comparison of bed levels across channels and intertidal platforms between scenarios with continued high mud supply and mangrove removal. Bed levels are extracted where the x-distance equals to 0.75 km.

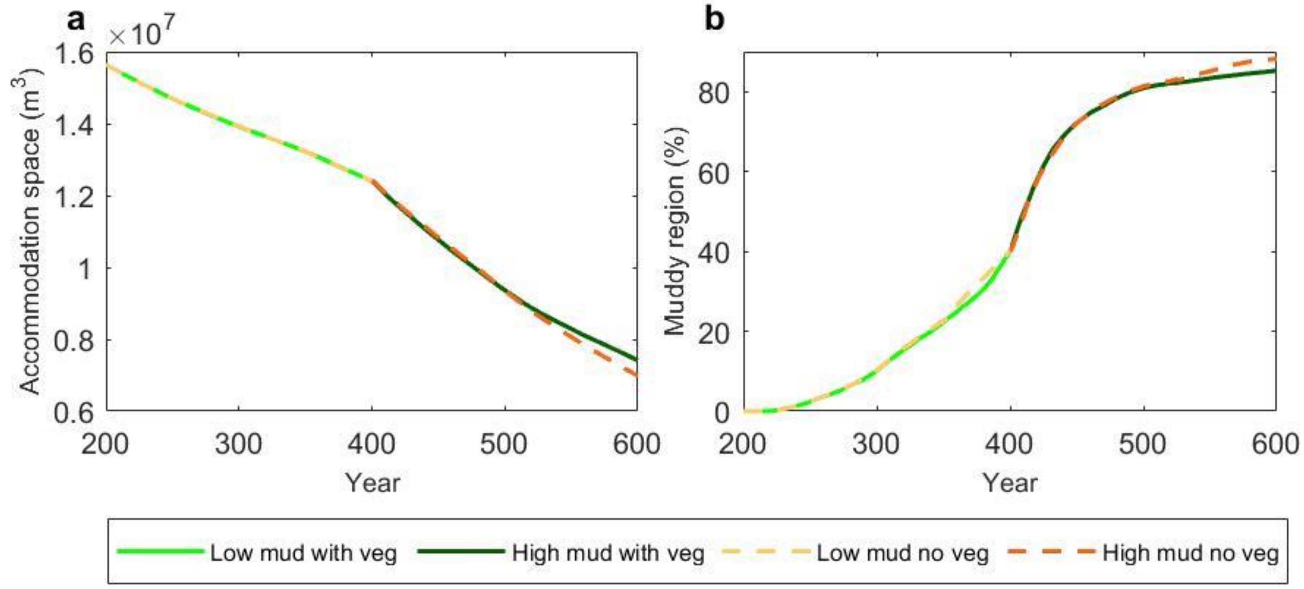

Fig. S5 Comparison of accommodation space and muddy region between scenarios with mangroves (solid lines) and without mangroves (dashed lines).

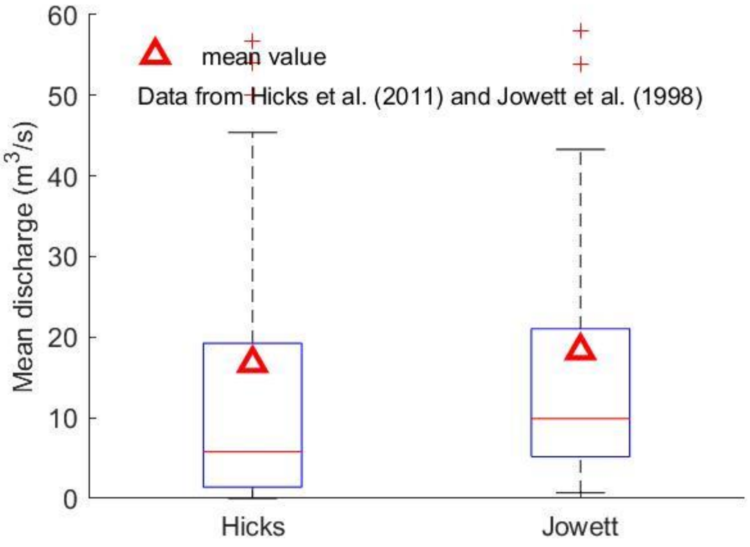

Fig. S6 Statistics of river discharge from main rivers in North Island, New Zealand. Data source is based on Hicks et al[1] and Jowett[2]. Red triangles represent their mean value, which equal to 18 m^3/s. The summarized data can be found in Supplementary Data 1.xlsx and Supplementary Data 2.xlsx.

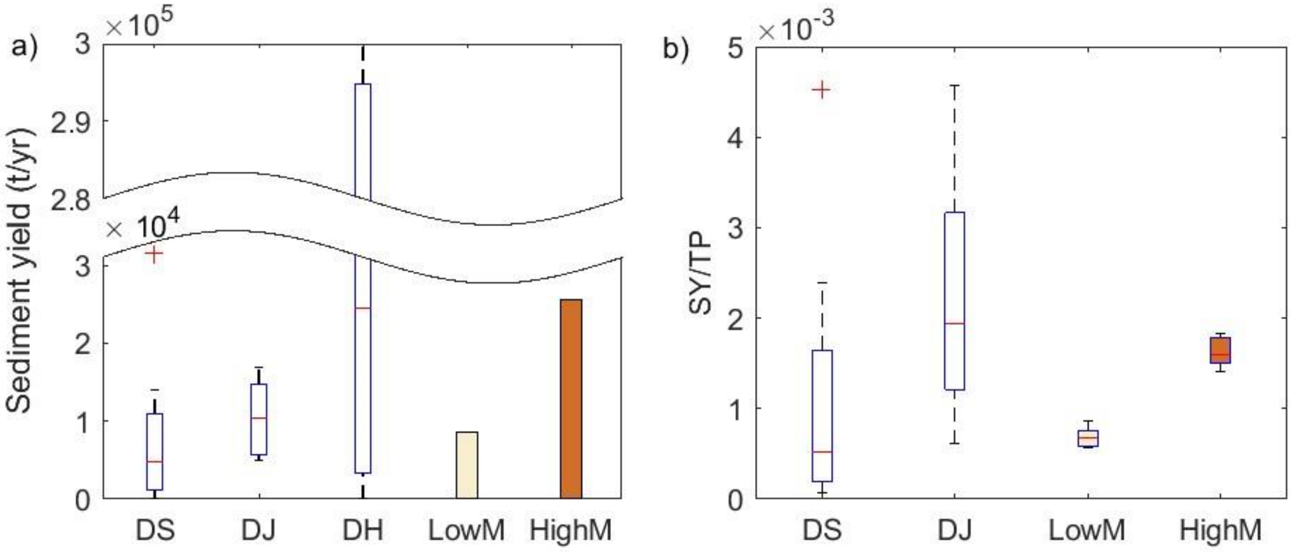

Fig. S7 Comparisons of sediment yield (a) and non-dimensional catchment sediment yield (b) derived from published data and model boundary settings. The published data for sediment yield is based on different resources, abbreviated as DS[3], DJ[4] and DH[1]. Corresponding tidal prism data are also available in the resources of DS and DJ. LowM and HighM represent low mud and high mud scenarios, respectively. The published data is summarized in Table S2 and Supplementary Data 1.xlsx. The modelled sediment yield (a) is calculated from a sum of the suspended sediment (i.e. mud) from all three rivers for a whole year. In subplot b), the simulated annual catchment sediment yield (SY) is the total sediment load from the three rivers, which contains both mud and potential import of sand (See Method). The tidal prism (TP) was calculated as the water discharged through the inlet during one tidal cycle. Here, suspended sediment supply for low mud and high mud scenarios are set to 5 mg/L and 15 mg/L, respectively.

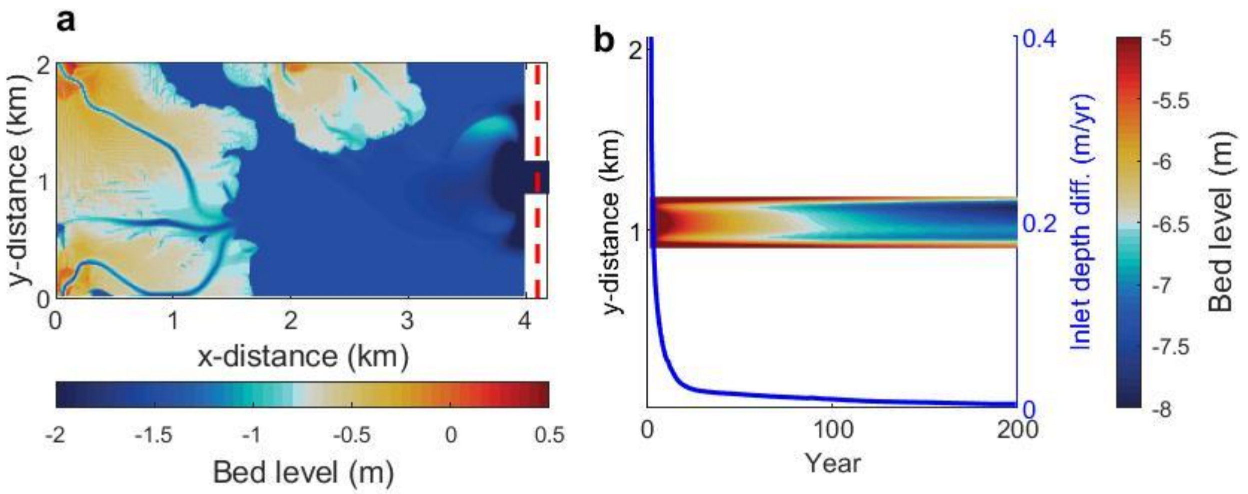

Fig. S8 Estuarine morphology and inlet depth change during spinup period. a) Estuarine morphology at the end of the spinup period. The red dashed line indicates the transect across the barrier in panel b). b) Bathymetry along the cross-sectional area at the inlet and annual difference of averaged inlet depth across the inlet section.

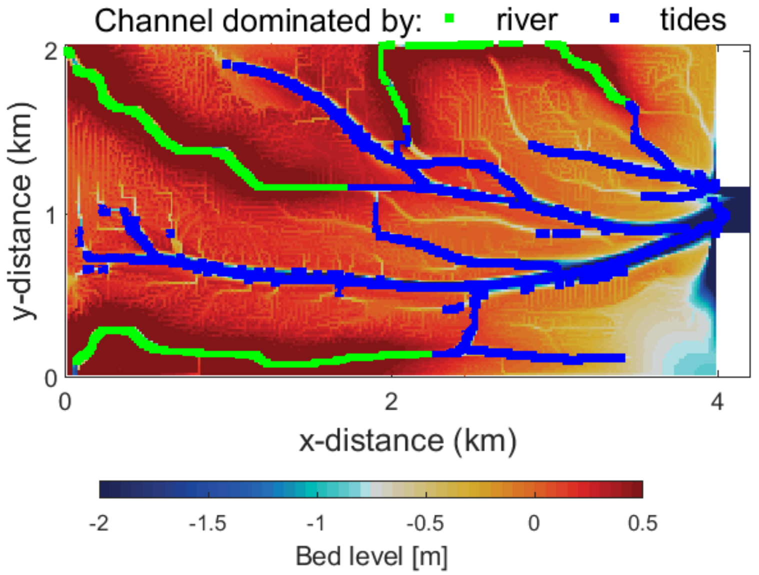

Fig. S9 Distribution of tide-dominated and river-dominated channels based on the scenario of Fig. 2d in the main text. When the flow is directed seaward even during the flood period, the channel is considered as river-dominated and marked in green (see example in Fig. S10). In contrast, when the flow is directed landward during the flood period and flow reversals occur, the channel is considered to be tide-dominated and marked in blue (see example in Fig. S11).

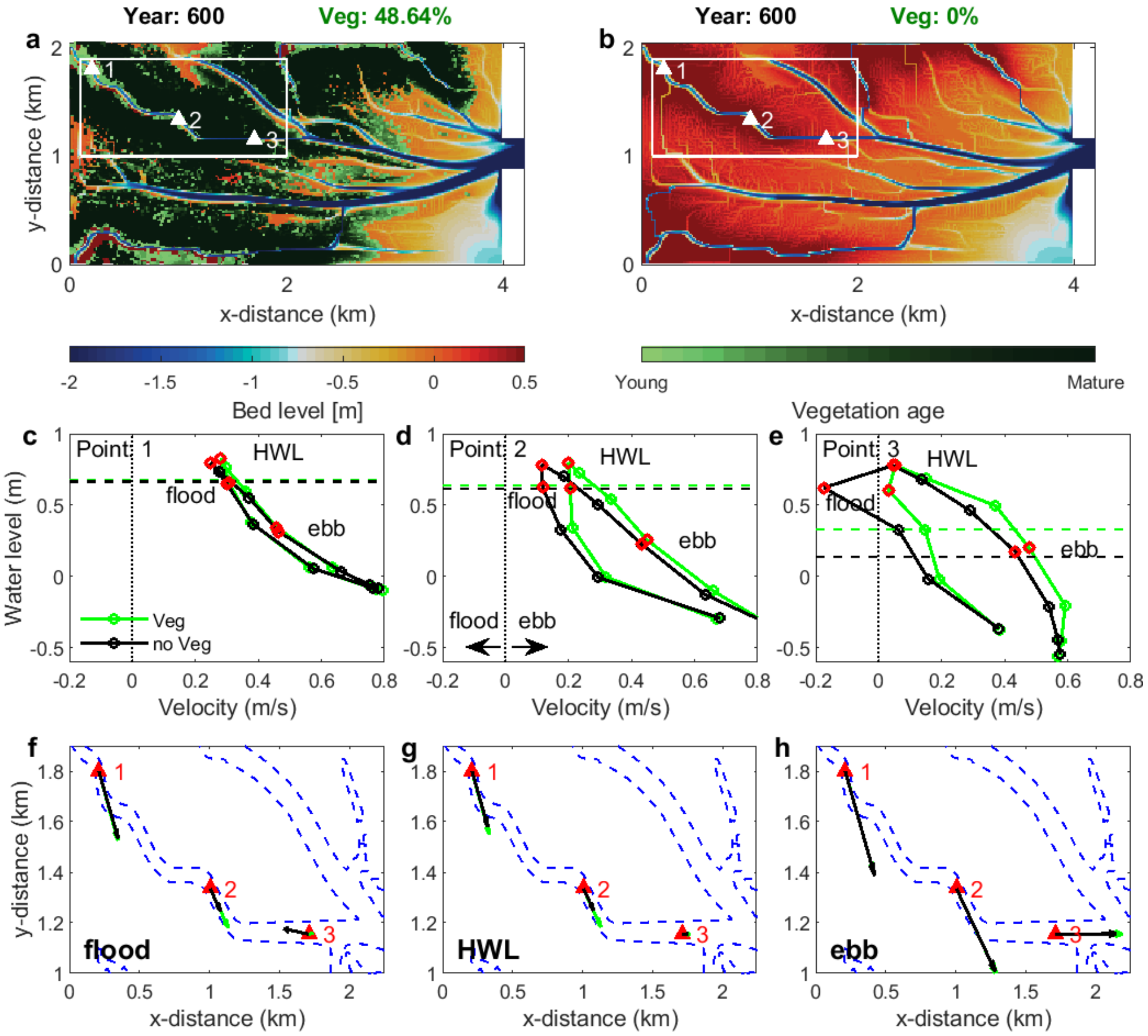

Fig. S10 Relation between water level and velocity within a river-dominated channel, with and without mangrove presence. a-b) Locations selected for comparisons. c-e) Tidal stage plots for the different locations along the channel. The horizontal green dashed lines indicate the vegetation elevation near the channel, and the elevation for the same area without vegetation is marked by a horizontal black dashed line f-h) Velocity vector at the selected points along the channel. The blue dashed lines delineate the channel band.

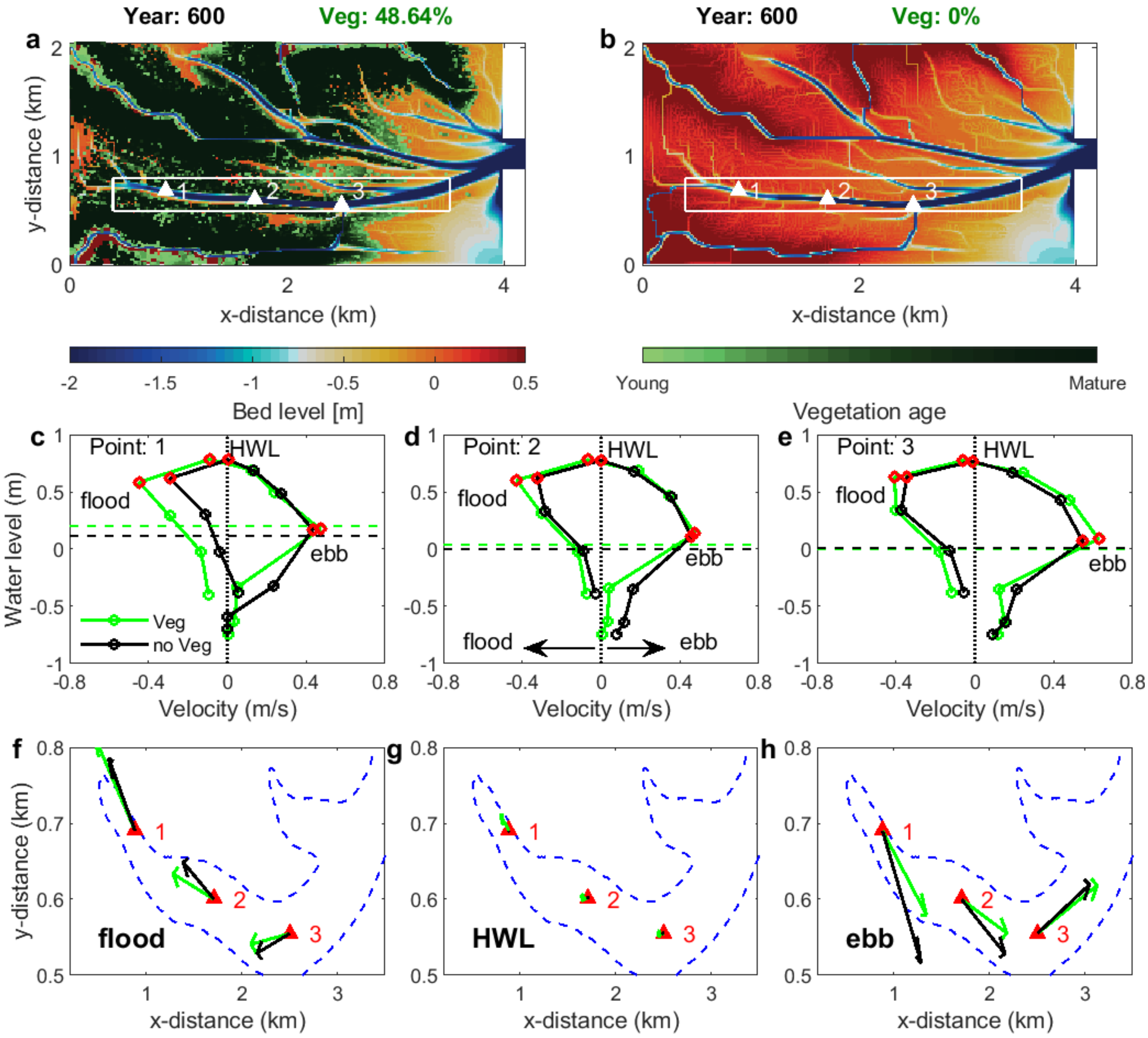

Fig. S11 Relation between water level and velocity within a tide-dominated channel, with and without mangrove presence. a-b) Locations selected for comparisons. c-e) Tidal stage plots for the different locations along the channel. The horizontal green dashed lines indicate the vegetation elevation near the channel, and the elevation for the same area without vegetation is marked by a horizontal black dashed line. f-h) Velocity vector at the selected points along the channel. The blue dashed lines delineate the channel band.

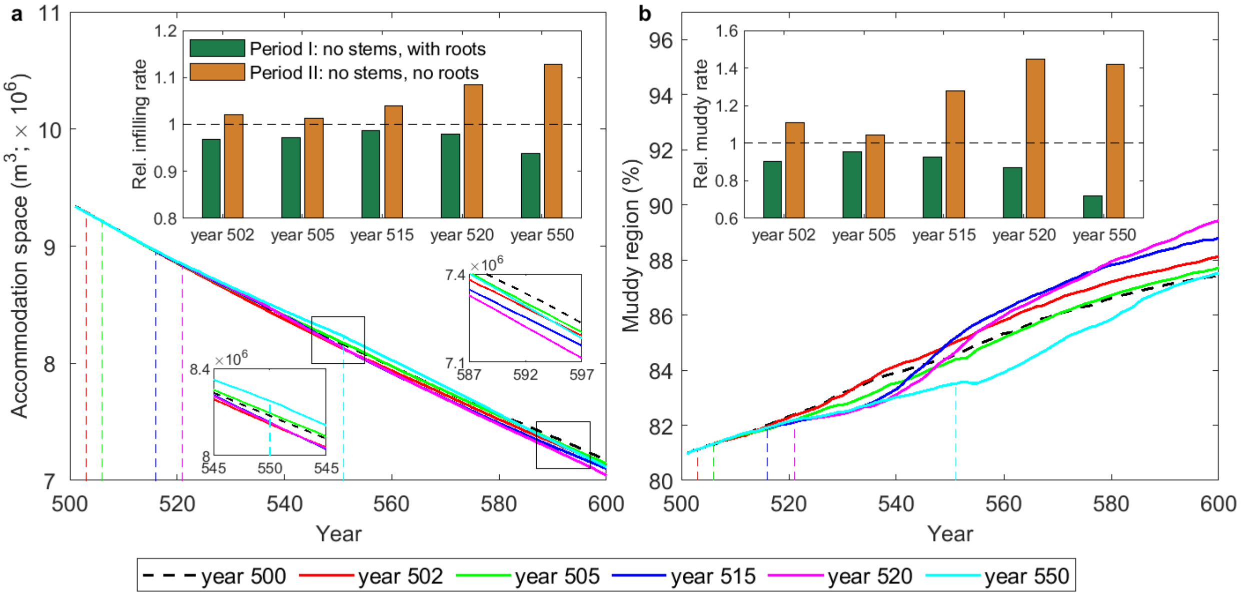

Fig. S12 Comparisons of changes in accommodation space (a) and muddy region (b) under different root persistence scenarios. The reference scenario applied in this comparison is based on Fig. 2k where both stems and roots are removed in year 500. As a comparison, different root persistence periods after mangrove removal, such as 2, 5, 15, 20 and 50 years, are shown as solid lines with different colors and has been indicated in Table S3. The inset plot in both panel (a) and (b) represents the relative (abbreviated as rel.) rate regarding the changes in accommodation space or muddy region fraction in two model periods differentiated by presence of roots. Specifically, a rate smaller than 1 means the examined scenario has a smaller infilling rate than the reference scenario in that period.

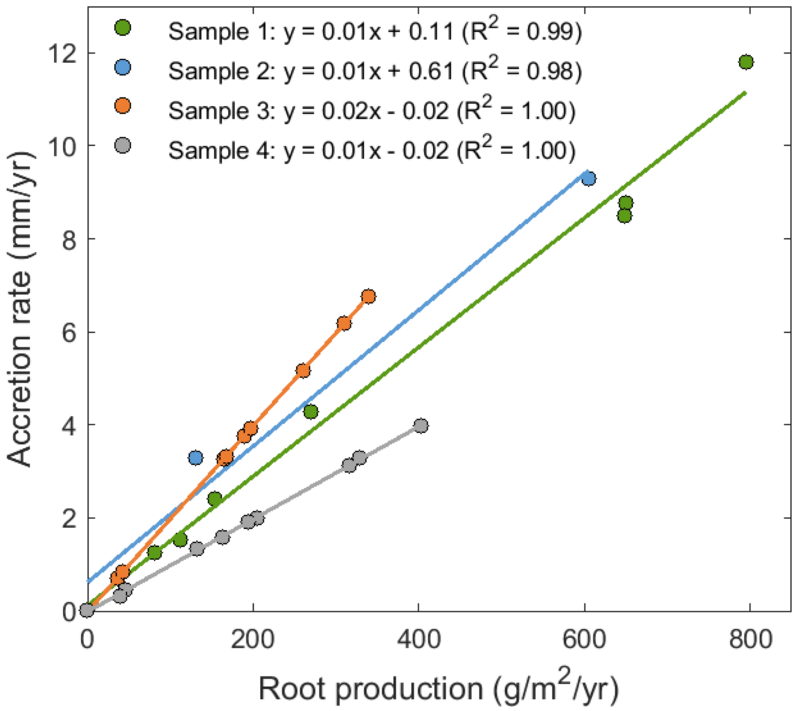

Fig. S13 Relations between the root production and surface accretion rates. Sample points are extracted from previous field observations summarized in Table S4. Linear regression lines are applied to each set of data samples to show a basic trend of accretion rate induced by different root accumulation.

Table S1. Dynamic vegetation model parameter settings.

Category	Parameter	Value/Description	Unit	Reference
**Vegetation parameters**	Initial stem diameter, D_0	0.8	cm	Bulmer et al[5] and Suyadi et al[6]
	Maximum root number, $N_{roots,max}$	1000	-	Young and Harvey[7] and Dahdouh-Guebas et al[8]
	Root diameter, D_{roots}	1	cm	van Maanen et al[9] and Xie et al[10]
	Root height, H_{roots}	15	cm	Horstman et al[11]
	Drag coefficient of roots, C_{Dr}	1	-	Xie et al[10] and Xie et al[12]
	Drag coefficient of stems, C_{Ds}	1.5	-	Xie et al[10] and Xie et al[12]
**Growth parameters**	Maximum stem diameter	18	cm	Bulmer et al[5] and Suyadi et al[6]
	Maximum tree height	320	cm	Bulmer et al[5] and Suyadi et al[6]
	Growth constant, G	36.88	cm/season	van Maanen et al[9] and Xie et al[10]
	Growth constant, b_2	31.94	-	van Maanen et al[9] and Xie et al[10]
	Growth constant, b_3	0.864	cm^{-1}	van Maanen et al[9] and Xie et al[10]
	Roots formula constant, k	0.5	-	Xie et al[10]

Table S2. Overview of estuarine infilling data in the estuaries of New Zealand from previous publications.

No.	Estuary	Tidal Prism (TP; 10^6 m^3)	Sediment yield (SY; t/yr)	Relative basin area above mean sea level (Amsl/Atot)	Sources
1	Whangateau	9.5	12492.64	0.33	Swales et al[3]
2	Matakana	2.8	6191.87	0.36	
3	Mahurangi	19.3	11867.23	0.33	
4	North Cove	0.9	153.42	0.20	
5	Bon Accord	4.4	491.42	0.08	
6	Puhoi	2.7	7474.91	0.60	
7	Waiwera	1.7	4872.28	0.82	
8	Ōrewa	1.8	1137.00	0.51	
9	Weiti	1.9	2542.17	0.58	
10	Ōkura	0.6	1137.00	0.63	
11	Waitemata	137.7	10708.77	0.22	
12	Tamaki	18.6	4789.58	0.18	
13	Whitford	21.5	14083.12	0.35	
14	Wairoa	5.8	31487.72	0.80	
15	Te Matuku	2.2	1238.62	0.36	
16	Awaawaroa	6.2	1744.36	0.13	
17	Putiki	5.6	849.90	0.25	
18	Whangapoua	8.54	6266.2	0.38	Hume and Herdendorf[13], Hicks and Shankar[14]
19	Wharekawa	3.1	8928.2	0.36	
20	Whangamatā	3.93	5048.8	0.21	

Table S3. Modelling scenarios testing the impacts of root persistence.

Run ID	Model period I (no stems, with roots)	Model period II (no stems, no roots)	Legend in the Fig. S12
1	-	501-600	year 500 (reference)
2	501-502	503-600	year 502
3	501-505	506-600	year 505
4	501-515	516-600	year 515
5	501-520	521-600	year 520
6	501-550	551-600	year 550

Table S4. Summary of field data on root accumulation and accretion rate.

No.	Root production ($g/m^2/yr$)	Accretion rate (mm/yr)	Site		Reference
1	649.36	8.77	Twin Cays, Belize and Rookery Bay, Florida		McKee[15]
2	269.26	4.28			
3	80.52	1.24			
4	648.05	8.49			
5	111.97	1.54			
6	153.92	2.4			
7	794.85	11.81			
8	130.2	3.3	Moreton Bay, Queensland		Lovelock et al[16]
9	604.8	9.3			
10	37	0.71	Fine roots at Twin Cays, Belize		McKee et al[17]
11	43	0.832			
12	189	3.753			
13	164	3.266			
14	260	5.172			
15	168	3.306			
16	197	3.915			
17	310	6.187			
18	339	6.755			
19	45	0.446	Coarse roots at Twin Cays, Belize		McKee et al[17]
20	39	0.324			
21	205	1.987			
22	316	3.123			
23	163	1.583			
24	132	1.339			
25	328	3.286			
26	194	1.906			
27	403	3.975			

Supplementary references

1. Hicks DM, *et al.* Suspended sediment yields from New Zealand rivers. *Journal of Hydrology (New Zealand)* **50**, 81-142 (2011).

2. Jowett IG. Hydraulic geometry of New Zealand rivers and its use as a preliminary method of habitat assessment. *Regul Rivers Res Manage* **14**, 451-466 (1998).

3. Swales A, *et al.* Potential future changes in mangrove-habitat in Auckland's east-coast estuaries. *NIWA Client Report: HAM2008-030 prepared for Auckland Regional Council*, (2009).

4. Jones HFE. Coastal sedimentation: what we know and the information gaps. In: *Environment Waikato Technical Report*). Waikato Regional Council (Environment Waikato) (2008).

5. Bulmer RH, Lewis M, O'Donnell E, Lundquist CJ. Assessing mangrove clearance methods to minimise adverse impacts and maximise the potential to achieve restoration objectives. *New Zealand Journal of Marine and Freshwater Research* **51**, 110-126 (2017).

6. Suyadi, Gao J, Lundquist CJ, Schwendenmann L. Aboveground Carbon Stocks in Rapidly Expanding Mangroves in New Zealand: Regional Assessment and Economic Valuation of Blue Carbon. *Estuar Coasts* **43**, 1456-1469 (2020).

7. Young BM, Harvey EL. A Spatial Analysis of the Relationship Between Mangrove (Avicennia marinavar.australasica) Physiognomy and Sediment Accretion in the Hauraki Plains, New Zealand. *Estuar Coast Shelf Sci* **42**, 231-246 (1996).

8. Dahdouh-Guebas F, Kairo J, De Bondt R, Koedam N. Pneumatophore height and density in relation to microtopography in the grey mangrove Avicennia marina. *Belgian Journal of Botany* **140**, 213-221 (2007).

9. van Maanen B, Coco G, Bryan KR. On the ecogeomorphological feedbacks that control tidal channel network evolution in a sandy mangrove setting. *Proc Math Phys Eng Sci* **471**, 20150115 (2015).

10. Xie D, *et al.* Mangrove diversity loss under sea-level rise triggered by bio-morphodynamic feedbacks and anthropogenic pressures. *Environmental Research Letters* **15**, 114033 (2020).

11. Horstman EM, Lundquist CJ, Bryan KR, Bulmer RH, Mullarney JC, Stokes DJ. The dynamics of expanding mangroves in New Zealand. In: *Threats to Mangrove Forests: Hazards, Vulnerability, and Management* (eds Makowski C, Finkl CW). Springer (2018).

12. Xie D, Schwarz C, Kleinhans MG, Zhou Z, van Maanen B. Implications of Coastal Conditions and Sea-Level Rise on Mangrove Vulnerability: A Bio-Morphodynamic Modeling Study. *Journal of Geophysical Research: Earth Surface* **127**, e2021JF006301 (2022).

13. Hume TM, Herdendorf CE. Factors Controlling Tidal Inlet Characteristics on Low Drift Coasts. *J Coast Res* **8**, 355-375 (1992).

14. Hicks D, Shankar U. Sediment from New Zealand rivers, NIWA chart, Miscellaneous Series.). NIWA (2003).

15. McKee KL. Biophysical controls on accretion and elevation change in Caribbean mangrove ecosystems. *Estuar Coast Shelf Sci* **91**, 475-483 (2011).

16. Lovelock CE, *et al.* Sea level and turbidity controls on mangrove soil surface elevation change. *Estuar Coast Shelf Sci* **153**, 1-9 (2015).

17. McKee KL, Cahoon DR, Feller IC. Caribbean mangroves adjust to rising sea level through biotic controls on change in soil elevation. *Global Ecol Biogeogr* **16**, 545-556 (2007).
