# Supplementary material for: Mangrove removal exacerbates estuarine infilling through landscape-scale bio-morphodynamic feedbacks
Source: Nat Commun. 2023 Nov 11;14:7310. doi: 10.1038/s41467-023-42733-1 (PMC10640651; doi:10.1038/s41467-023-42733-1)
Supplement: Supplementary file 3 — Description of Additional Supplementary Files [file 41467_2023_42733_MOESM3_ESM.docx]

**Description of Additional Supplementary Files**

**File name:** Supplementary Data 1

**Description:** Summarized river flow and sediment yield data of 148 New Zealand rivers.

**File name:** Supplementary Data 2

**Description:** Summarized river flow and width data of 73 New Zealand rivers.

**File name:** Supplementary Data 3

**Description:** Mangrove coverage fraction (relative to estuary area) in 2020, tidal prism and sediment yield data of three representative estuaries in New Zealand.

**File name:** Supplementary Data 4

**Description:** Tidal level information of three representative estuaries in New Zealand.
